# Prostaglandin E2 Exerts Biphasic Dose Response on the PreBötzinger Complex Respiratory-Related Rhythm

**DOI:** 10.3389/fncir.2022.826497

**Published:** 2022-05-20

**Authors:** Jan Philipp Reising, Wiktor S. Phillips, Naify Ramadan, Eric Herlenius

**Affiliations:** ^1^Department of Women’s and Children’s Health, Karolinska Institutet, Stockholm, Sweden; ^2^Astrid Lindgren Children’s Hospital, Karolinska University Hospital, Stockholm, Sweden

**Keywords:** preBötzinger complex (preBötC), prostaglandin E2 (PGE2), inflammation, neural networks, respiratory – mechanics

## Abstract

Inflammation in infants can cause respiratory dysfunction and is potentially life-threatening. Prostaglandin E2 (PGE2) is released during inflammatory events and perturbs breathing behavior *in vivo*. Here we study the effects of PGE2 on inspiratory motor rhythm generated by the preBötzinger complex (preBötC). We measured the concentration dependence of PGE2 (1 nM-1 μM) on inspiratory-related motor output in rhythmic medullary slice preparations. Low concentrations (1–10 nM) of PGE2 increased the duration of the inspiratory burst period, while higher concentrations (1 μM) decreased the burst period duration. Using specific pharmacology for prostanoid receptors (EP1-4R, FPR, and DP2R), we determined that coactivation of both EP2R and EP3R is necessary for PGE2 to modulate the inspiratory burst period. Additionally, biased activation of EP3 receptors lengthened the duration of the inspiratory burst period, while biased activation of EP2 receptors shortened the burst period. To help delineate which cell populations are affected by exposure to PGE2, we analyzed single-cell RNA-Seq data derived from preBötC cells. Transcripts encoding for EP2R (*Ptger2*) were differentially expressed in a cluster of excitatory neurons putatively located in the preBötC. A separate cluster of mixed inhibitory neurons differentially expressed EP3R (*Ptger3*). Our data provide evidence that EP2 and EP3 receptors increase the duration of the inspiratory burst period at 1–10 nM PGE2 and decrease the burst period duration at 1 μM. Further, the biphasic dose response likely results from differences in receptor binding affinity among prostanoid receptors.

## Introduction

Infections in neonates increase both the frequency of apneas and the risk of death due to cardiorespiratory failure (Bruhn et al., 1977; Naeye, 1979; Schulte et al., 1982; Fanaroff et al., 1998; Kamaluddeen et al., 2009; Stock et al., 2010; Schiller et al., 2011; Schroeder et al., 2013). The innate inflammatory response is sufficient to disturb breathing even in the absence of an infection. Inflammatory pathway stimulants like lipopolysaccharide (LPS) and Interleukin-1β (IL-1β) induce apneas and dampen the ability to autoresuscitate following acute anoxia (Stoltenberg et al., 1994; Frøen et al., 2000; Olsson et al., 2003, Hofstetter and Herlenius, 2005; Lorea-Hernández et al., 2016; Morrison et al., 2020).

Prostaglandin E2 (PGE2) is released during inflammatory events like infection and induces apneas and irregular breathing *in vivo* (Olsson et al., 2003; Hofstetter et al., 2007). However, the effects of PGE2 are less clear when studied in isolated regions of the brainstem respiratory circuitry *ex vivo*. Reduced preparations of the rodent brainstem continue to produce respiratory-like motor rhythms *in vitro* and have been used as an experimental model to more thoroughly investigate how PGE2 modulates the central control of breathing behavior (Suzue, 1984; Smith et al., 1991; Hofstetter and Herlenius, 2005). In mice, applying 10–50 nM PGE2 to *en bloc* brainstem spinal cord preparations decreases the frequency of rhythmic inspiratory-related motor output, recapitulating observations made *in vivo* (Olsson et al., 2003; Hofstetter et al., 2007; Forsberg et al., 2016). However, in further-reduced preparations, like medullary slices containing the preBötC, 2 μM PGE2 increases the frequency of inspiratory-related motor output (Koch et al., 2015). The disparity between these two findings might simply reflect differences in the experimental tissue preparation. In the rhythmic slice preparation, the preBötC is deafferented from rostral brainstem populations that normally participate in respiratory pattern generation and modulation. These lost afferents might otherwise provide neuromodulatory feedback that is necessary for PGE2 to depress breathing. On the other hand, the concentration of PGE2 used in acute slice recordings was approximately 40-fold greater than other experiments done in the *en bloc* brainstem spinal cord or *in vivo.* Opposing changes in the frequency of inspiratory-related activity could therefore also be explained by a concentration-dependent mechanism—a biphasic dose response.

Prostaglandin E2 primarily binds to four receptors of the prostanoid family, prostaglandin receptors 1 to 4 (EP1-4R) (Narumiya et al., 1999; Woodward et al., 2011), which have different binding affinities for PGE2 and couple to various G-protein subunits (Table 1). Genetic deletion of EP3 receptors blunts ventilatory responses to inflammation, but the contribution of other prostanoid receptors has not been similarly tested (Siljehav et al., 2014; Forsberg et al., 2016, 2017). A biphasic response to PGE2 has been described previously in other systems, where different prostanoid receptors can mediate the responses at different concentration ranges of PGE2 (Kather and Simon, 1979; Raisz and Fall, 1990; Sergeeva et al., 1997; Yamato et al., 2005; Philipose et al., 2010; Zhang and Wang, 2014; Mawhin et al., 2015; Pasterk et al., 2015). We hypothesized that more than one prostanoid receptor subtype can modulate the frequency of inspiratory-related motor output, and that biased activation of these receptors can either stimulate or depress inspiratory frequency. The dissociation constants of EP1 and EP2 receptors for PGE2 are two orders of magnitude higher than EP3 and EP4 receptors. Therefore, concentrations of PGE2 in the 1–10 nM range ought to saturate EP3/EP4 receptors, while only partially binding EP1/2 receptors. We expected that activation of high affinity receptors like EP3 or EP4 would depress inspiratory frequency, while the additional recruitment of lower affinity receptors, like EP1 or EP2, would stimulate inspiratory frequency. We measured inspiratory motor output in acute medullary slices containing the preBötC and found that PGE2 elicited a biphasic concentration-dependent change in burst period duration. We also identified EP2 and EP3 receptors as the main effectors of PGE2-induced modulation of the inspiratory rhythm.

**TABLE 1:** Overview of prostanoid receptors and pharmacology.

Receptor	Affinity (nM)	Antagonist	Agonist	Signaling
EP1R	25	GW-848867X	11-deoxy-PGE1	↑ Ca^2+^
EP2R	13	PF-04418948	Butaprost	G_*s*_
EP3R	0.33	DG-041	Sulprostone	α/β: Gi γ: G_*i*/_G_*s*_
EP4R	0.59	MK-2894	–	G_*s*_
FP	119	AL-8810	–	G_*q*_
DP2	307	Setipiprant	–	G_*i*_, ↑ Ca^2+^

*Reviewed in Abramovitz et al. (2000).*

## Materials and Methods

### Animals

Unless otherwise stated, all experiments were performed on outbred CD-1 mice (Charles River, Germany). Mice in which the Ptger3 gene (EP3R) was selectively deleted had a C57BL/6J background (Fabre et al., 2001). All mice were reared by their mothers under standardized conditions with a 12:12-h light-dark cycle. Food and water were provided *ad libitum*. The studies were performed in accordance with European Community Guidelines and approved by the regional Ethics Committee. The animals were reared and kept at the Department of Comparative Medicine, Karolinska Institutet, Stockholm, Sweden.

### Slice Preparation

Mice were sedated on postnatal day (P) 2–4 using 5% Vetflurane (Virbac Animal Health Ltd. Bury St. Edmunds, Suffolk, United Kingdom) dispersed by an anesthesia unit (AgnTho’s AB, Univentor 410). After decapitation, tissue was placed in ice-cold artificial cerebrospinal fluid (ACSF) containing (in mM): 25 D-glucose, 2.5 KCl, 1.25 NaH_2_PO_4_, 12 N-acetyl–L-cystein, 147 glycerol, 3 ethyl pyruvate, 20 HEPES (pH 7.4 at 4°C), 5 sodium ascorbate, 30 NaHCO_3_, 10 MgSO_4_, 0.5 CaCl_2_. Final osmolarity was 300–320 mOsm after bubbling with 95% O_2_/5% CO_2_. The brainstem was removed and attached (cyanoacrylate glue) dorsal-side down to an angled agar block (tilted 20° from vertical). The agar block was then attached to the stage of a vibrating microtome (7000smz-2, Campden Instruments, Loughborough, United Kingdom). Serial transverse slices of 200–400 μm thickness were taken starting from the rostral end of the brainstem until the opening of the 4th ventricle was reached. Subsequent trim slices (100–200 μm thickness) were cut at low speeds (<50 μm/s advance) until landmarks associated with the preBötC could be seen (Ruangkittisakul et al., 2014). For *in vitro* recordings, rhythmic slices were cut 650 μm thick, with the preBötC exposed on the rostral surface of the slice. For single-cell RNA-Seq samples, slices were cut 300 μm thick, with the preBötC centered in the slice.

### Electrophysiology

Transverse slices were transferred to a recording chamber (BSC1, Scientific Systems Design Inc., Mississauga, Canada) and perfused with recording ACSF (bubbled with 95% O_2_/5% CO_2_, 26°C, 300–320 mOsm, pH 7.3–7.4). Recording ACSF, containing (in mM): 25 D-glucose, 9 KCl, 1.25 NaH_2_PO_4_, 1 sodium ascorbate, 26 NaHCO_3_, 115 NaCl, 1 MgCl_2_, 1.5 CaCl_2_. A suction electrode pipette with ca. 70–100 μm opening (GC120-15, borosilicate glass capillaries, 1.2 mm O.D. × 0.69 mm I.D., Clark Electromedical Instruments, Reading, United Kingdom) was gently attached to the hypoglossal (XII) rootlets, which were retained in the transverse slice. The extracellular recording signal was amplified with a differential AC Amplifier (Model 1700, A-M Systems, Sequim, United States). The signal was filtered (low-cut 300 Hz, high-cut 1,000 Hz, Gain 10,000×) and recorded with a digitizer (Digidata 1320A, Molecular Devices, San Jose, CA, United States) at 20,000 samples/s.

Slices were perfused with ACSF for 20–40 min prior to recording a 10-min baseline. Drug stocks were added to recording ringer and sequentially washed into the chamber using an automated pinch valve system (PC-16 controller, Bioscience Tools, San Diego, CA, United States). Slices were exposed to drugs for 20 min before moving on to the next drug. However, only the last 10 min of each exposure epoch were analyzed to allow for stabilization of the rhythm. The following drugs were used: EP1 antagonist GW848867X (Cayman Chemicals, Ann Arbor, MI, United States, 50 nM), EP2 Antagonist PF-04418948 (Cayman Chemical, 500 nM), EP3 antagonist DG041 (Tocris, Bristol, United Kingdom, 40 nM), EP4 antagonist MK2894 (APExBIO, Houston, TX, United States, 100 nM), Prostaglandin E2 (Sigma-Aldrich, St. Louis, MO, United States, 1 nM-1 μM), Prostaglandin F receptor antagonist AL-8810 (Cayman Chemical, 1 μM), Prostaglandin D2 receptor antagonist Setipiprant (Cayman Chemical, 500 nM), Non-selective EP2/3/4 agonist 11-deoxy PGE1 (Cayman Chemical, 10 nM), EP2R agonist Butaprost (Cayman Chemical, 5 μM), EP3 receptor agonist Sulprostone (Cayman Chemical, 1 μM).

Raw data from XII rootlet recordings was analyzed using custom software written in Python. In short, XII recordings were resampled at 1/10 rate and artifacts were excluded using a Hampel filter with a 0.5 s window and a threshold of 5 standard deviations. Bursts were defined as peaks of minimum prominence of 0.2 and distance of 15 s. Results were manually checked for false positives. XII burst area was implemented as a leaky integrator with a decay constant of 0.98. Burst characteristics were calculated for each burst individually and subsequently averaged for each sample. If percentages are given, values were normalized to the mean of the baseline period for the given variable. Two-sided Smirnov-Grubb’s test was performed to exclude outliers with *p* < 0.05. Multimodality was tested using the Hartigan’s Dip test (Hartigan and Hartigan, 1985).

Statistics for rootlet recordings are the result of General Estimating Equations (GEE) performed with a custom R script using the “geepack” and “emmeans” packages. GEE was chosen as an alternative to an ANOVA, since the assumption of homogeneity of variances was not satisfied. This can be explained by the increase in variability after addition of PGE2, leading to different variances between the experimental groups. In short, a Gaussian GEE model was fitted to the data set and subsequently Bonferroni adjusted contrasts were calculated, if permitted by the results of the GEE model.

### Single-Cell Transcriptomics Sample Preparation

Outbred CD-1 littermates (age P4) were dissected on the same day, using the methods described in Slice Preparation. All procedures were carried out under aseptic conditions. We cut slices of 300 μm thickness, with the preBötC approximately centered in the slice according to anatomical landmarks (Ruangkittisakul et al., 2014). Using a sterilized metal tool (NIH Style Neuro Punches, Agnthos, Lidingö, Sweden), circular punchouts (ø 690 μm) were cut from the ventral-lateral border on both sides of each slice, centered over the approximate location of the preBötC in the transverse plane. After extraction, the punchouts were immediately placed into dissociation ACSF containing (in mM): 25 D-glucose, 2.5 KCl, 1.25 NaH_2_PO_4_, 12 N-acetyl-L-cysteine, 3 ethyl pyruvate, 20 HEPES, 5 sodium ascorbate, 30 NaHCO_3_, 10 MgSO_4_, 0.5 CaCl_2_, and 0.1 U/μL RNase inhibitor (RNasin Plus, Promega, Madison, WI, United States). In addition, we added 5% w/v D-trehalose dihydrate (VWR Chemicals, Radnor, PA, United States) to improve cell viability (Saxena et al., 2012). The final osmolarity of the dissociation ACSF was 300–320 mOsm and pH was 7.3–7.4 after bubbling with 95% O_2_/5% CO_2_. We enzymatically dissociated the cells by adding 1 mg/mL pronase (Roche, Basel, Switzerland) to the dissociation buffer. Dissociation was carried out in silanized glass conical tubes at room temperature (21–22°C) for 25 min with periodic agitation of the solution. During this time, the head space of the conical tube was continuously gassed with 95% O_2_/5% CO_2_. We then titrated the tissue with silanized glass Pasteur pipettes, which had been fire polished to an inner diameter of 200, 400, or 800 μm. Further protease action was then inhibited by addition of 1% fetal bovine serum (FBS) to the dissociation buffer. Additionally, we added a fixable fluorescent viability indicator to stain dead cells (Live-or-Dye NucFix Red, Biotium, Fremont, CA, United States). Cells were incubated on ice in the FBS/dye-containing solution for 5 min and then centrifuged at 300 × *g* for 5 min. The cell pellet was resuspended in ice-cold dissociation ACSF containing 2% FBS that had been pre-equilibrated with 95% O_2_/5% CO_2_. Finally, we cryopreserved the cells in methanol (Alles et al., 2017). In brief, methanol chilled to −20°C was added dropwise to the resuspended cells (final concentration 80% methanol). Methanol-fixed samples were allowed to incubate at 4°C for at least 15 min before being transferred to a −80°C freezer for storage. Samples were stored for less than 1 week before subsequent library preparation.

### Single-Cell Transcriptomics Library Generation, Sequencing, and Data Processing

Single-cell transcriptomic libraries were generated with the STRT-seq-2i method (Hochgerner et al., 2017). First, methanol-fixed samples were pooled into a single 15 mL conical tube and centrifuged at 2,000 × *g* for 10 min. Cells were resuspended in a solution of ice-cold PBS without Ca^2+^or Mg^2+^, including 20 μg/mL Hoechst 33342 (Merck), 2% bovine serum albumin (Merck, Kenilworth, NJ, United States) and 0.5 U/μL RNase inhibitor (RNasin Plus, Promega). Cells were washed a second time, counted, and then diluted to a concentration of 20 cells/μL and less than 0.1% BSA. The cells were dispensed via limiting dilution (iCell8 MSND, Wafergen, Fremont, CA, United States) into a 9600 microwell array (Wafergen). The chip was imaged on a microscope equipped with epifluorescence and screened via software detection (CellSelect, Wafergen) for empty wells, doublets (via Hoechst fluorescence), and dead cells (via red viability stain). Reagents for library preparation were only dispensed to wells called as viable singlets. All remaining library preparation, quality control, and tagmentation steps were performed as described in Hochgerner et al. (2017) Final libraries were sequenced on an Illumina HiSeq 2500, 50 bp single-end reads. Raw sequencing data was pre-processed as previously described (Hochgerner et al., 2017). In brief, UMIs containing any bases of Phred score <17 were rejected. Reads with less than 3 Gs following the 6 bp UMI were filtered out. After trimming poly-A tails, only reads with at least 25 remaining bases were kept. Filtered reads were mapped and demultiplexed with zUMIs (Parekh et al., 2018). Detected barcodes were filtered according to the barcode combinations dispensed during library tagmentation. Total reads per cell were adaptively downsampled to within three times the median absolute deviation.

### Single-Cell Transcriptomics Analysis

The raw count matrix was further processed and analyzed using “Scater” and “Seurat” libraries in R (McCarthy et al., 2017; Hao et al., 2021). Cells expressing less than 1,000 genes, and genes expressed in fewer than 10 cells were excluded from analysis. Of the remaining cells, we eliminated outliers with log-library sizes and percent mitochondrial reads exceeding three times the median absolute deviation. Counts were normalized and scaled using “SCTransform” (Hafemeister and Satija, 2019). We removed doublets using “DoubletFinder” (McGinnis et al., 2019). Finally, the data was dimensionally reduced via PCA, and the first 20 PCs were used to cluster cells via the graph-based Leiden algorithm (Traag et al., 2019). The data was also dimensionally reduced to a 2D visualization via uniform manifold approximation and projection (UMAP) using the first 20 PCs (McInnes et al., 2020). We tested for differential expression with a non-parametric Wilcoxon rank sum test. Resulting *p*-values were adjusted with a Bonferroni correction, using the total number of genes in the count matrix. We tested DE only on genes which were expressed by at least 10% of the cells in a cluster and with log2 fold-change between groups greater than 0.1. We compared each cluster to all other cells in the data (“FindAllMarkers” function in “Seurat”). Non-neuronal clusters were annotated by differential expression for astrocytes, microglia, endothelial cells, oligodendrocytes, and oligodendrocyte precursor cells. We also compared differential expression in each of the remaining clusters with respect to all cells in the 5 non-neuronal clusters. Differential expression values are reported as the log2 fold-change in average expression between the comparison groups, followed by the percentage of cells expressing transcripts in the first group versus percentage of cells in the comparison group expressing gene transcripts.

## Results

### Prostaglandin E2 Elicits Concentration-Dependent Response in the PreBötzinger Complex Burst Period

**FIGURE 1:**
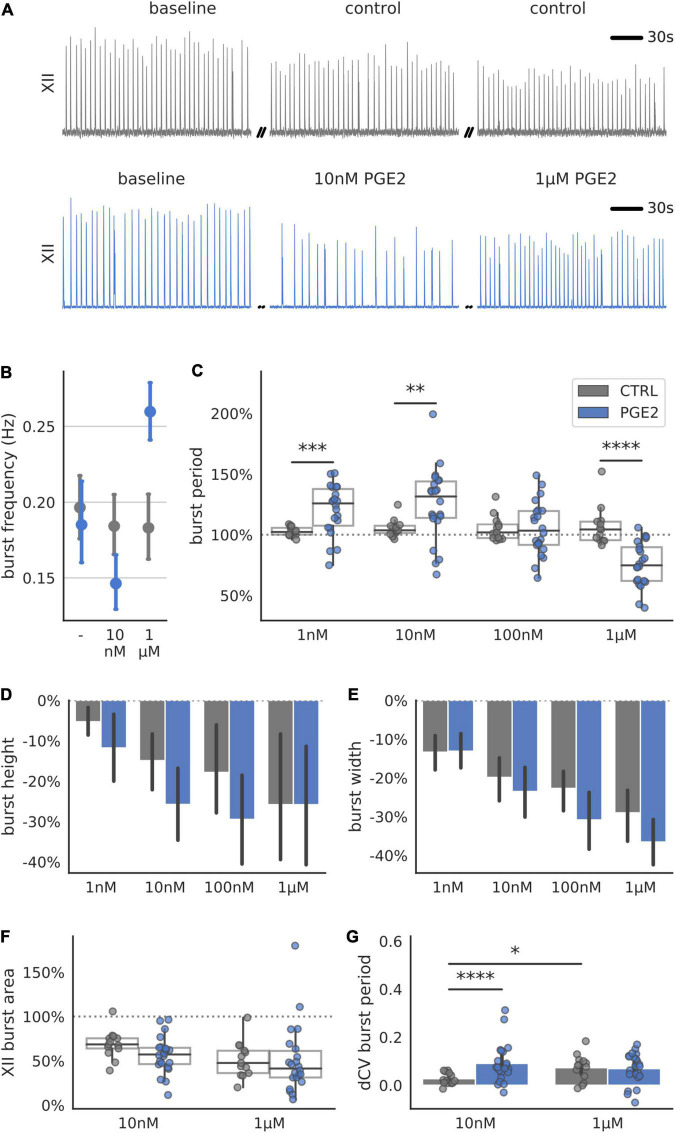
Respiration-related XII motor nucleus output of transverse preBötC slices during increasing levels of PGE2. **(A)** Examples of integrated hypoglossal motor output (XII) of control and PGE2 exposed slices (150 s shown per condition). **(B)** Biphasic response of the burst frequency (Hz) to increasing concentrations of PGE2. **(C)** Normalized mean burst period, defined as time between subsequent bursts and normalized to baseline, is significantly different from untreated controls at 1 nM (+18% above control, *p* < 0.001), 10 nM (+20% above control, *p* < 0.01) and 1 μM PGE2 (–31% below control, *p* < 0.0001). **(D)** Burst height, defined as the maximum during the burst, and **(E)** burst width gradually decrease over time for control and PGE2 treated slices. **(F)** Mean normalized XII burst area, defined as the integral of the burst, decreased for control and PGE2 slices. **(G)** Regularity of respiratory rhythm quantified using the difference in Coefficient of Variation (CV) of the burst period increased for slices treated with 10 nM PGE2 compared to control (+0.06, *p* < 0.0001) (*p < 0.05, **p < 0.01, ***p < 0.001, ****p < 0.0001).

We measured the dose response of PGE2 on inspiratory-related motor activity via extracellular recordings taken from hypoglossal nerve rootlets in rhythmically active transverse slice preparations containing the preBötC (Figure 1A). Lower concentrations of PGE2 (1–10 nM) increased the mean duration of the inspiratory burst period relative to baseline values (+21% above baseline in 1 nM PGE2, +25% above baseline in 10 nM PGE2), which was significantly different from the mean burst period measured in untreated control slices (Figures 1B–C). Additionally, the duration of the burst period was more irregular in 10 nM PGE2 compared to control slices (Figure 1G). In 1 μM PGE2, the mean duration of the burst period decreased compared to baseline values (−24% below baseline in 1 μM PGE2), which was also significantly different from untreated control slices (Figure 1C).

In all the recordings from PGE2-treated and control slices, burst amplitude and burst width gradually decreased over the duration of the recording (Figures 1D,E). However, this rundown was consistent and not statistically different between control and treatment groups. Similarly, there was no difference in the XII burst area (Figure 1F). These results suggest that PGE2 primarily influences the timing and regularity of inspiratory-related burst generation, but not the shape (i.e., pattern) of inspiratory motor output. However, changes in the timing of inspiratory bursts measured on XII rootlets could also result if synchronized rhythmic activity in the preBötC fails to propagate to downstream motor nuclei.

### Burst Period With and Without Exposure to Prostaglandin E2 Is Unimodally Distributed

**FIGURE 2:**
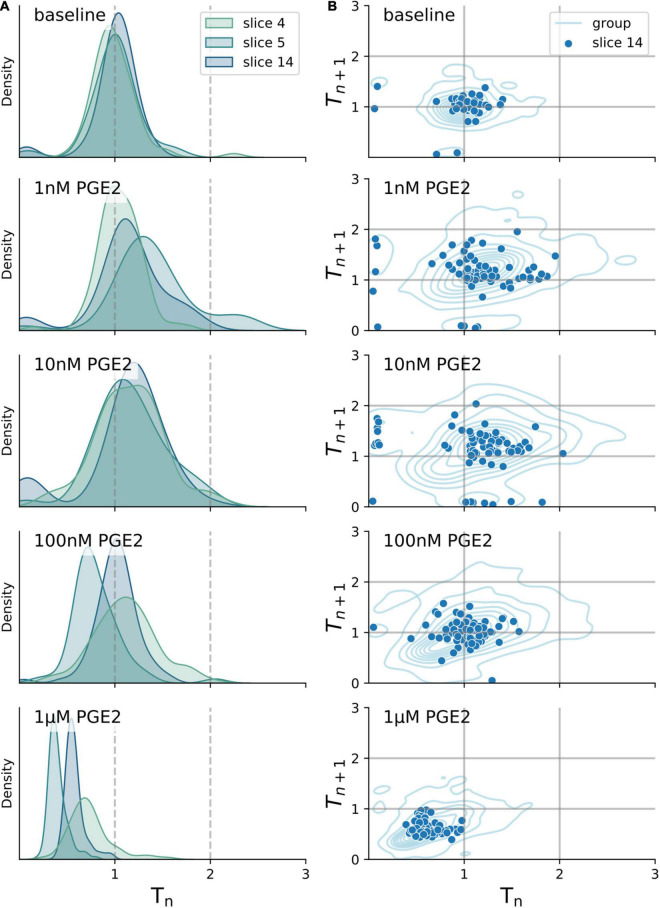
Burst period stays unimodally distributed after exposure to increasing levels of PGE2. **(A)** Distribution of burst period (T_*n*_) normalized to baseline of three exemplary transverse slices after exposure to 1 nM–1 μM PGE2. **(B)** Correlation of T_*n*_ to subsequent burst period (T_*n+*1_) normalized to baseline for a single exemplary transverse slice (dots) and combined periods of all slices (lines). All distributions shown are unimodal as tested for by the Hartigan’s Dip test (*p* > 0.05).

To determine whether PGE2 modulates rhythm- or pattern-generating cell populations in the preBötC, we further analyzed the distribution of individual burst periods in each slice recording (Figure 2). Burstlet theory posits that inspiratory rhythm and pattern generation are separable processes that arise from the activity of specialized neuronal subpopulations within the preBötC (Kam et al., 2013; Cui et al., 2016). If PGE2 predominately modulates the excitability of pattern-generating neurons, we would expect to see the burst period distribution become multimodal. The additional peaks in a multimodal distribution reflect dropout events where burstlets fail to elicit network-wide bursts that propagate to the XII nucleus. However, if PGE2 predominately modulates the rhythm-generating population, we would expect to see a shift or broadening of the burst period distribution that is not multimodal. We therefore tested whether the burst period distribution in our recordings became multimodal after exposure to PGE2. The distribution at baseline and after exposure to PGE2 of all bursts, grouped by experimental condition, was unimodal (Hartigan’s Dip test: *p* > 0.05, Figure 2). Aggregating measurements across multiple slices into a single distribution might mask effects on the individual slice level, so we further checked for multimodality in each slice and condition independently. The burst period distributions in 20 out of 21 individual slices were unimodal. The modes of the multimodal slice recording were centered around 0.93 ± 0.08 and 1.43 ± 0.16 at 100 nM PGE2 (Hartigan’s Dip test: *p* < 0.05) and 0.62 ± 0.16 and 1.01 ± 0.06 at 1 μM PGE2 (Hartigan’s Dip test: *p* < 0.05). These results suggest that PGE2 modulates inspiratory rhythm generation (i.e., timing of burstlets) rather than pattern generation (burst generation and downstream propagation).

### EP2R and EP3R Are Necessary to Mediate Prostaglandin E2 Responses

**FIGURE 3:**
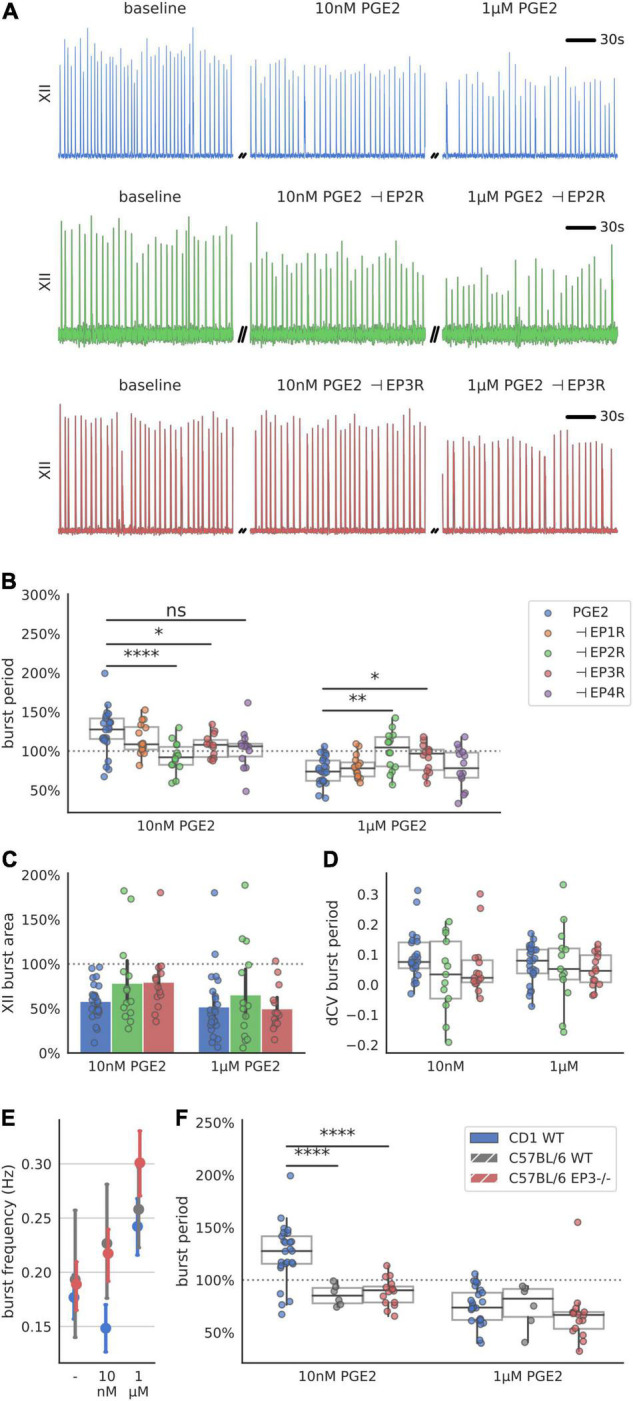
Respiration-related XII motor nucleus output after incubation with a prostanoid antagonist and exposure to 10 nM and 1 μM PGE2. **(A)** Examples of integrated hypoglossal motor output (XII) of slices exposed to PGE2 with and without inhibiting EP2R (500 nM PF-04418948) and EP3R (40 nM DG-041) (150 s shown per condition). **(B)** Inhibition of EP2R leads to a decrease of burst period at 10 nM (–8% below baseline, –33% below uninhibited controls, *p* < 0.00001 vs. controls) and no change at 1 μM (±0% above baseline, +25% above uninhibited controls, *p* < 0.01 vs. controls). Inhibition of EP3R leads to an increase at 10 nM (+7% above baseline, –18% below uninhibited controls, *p* < 0.05 vs. controls) and decrease at 1 μM (–9% below baseline, +16% above uninhibited controls, *p* < 0.05). **(C)** Antagonizing either EP2R or EP3R prior to exposure to PGE2 does not change XII burst area. **(D)** Change in CV of the burst period after exposure to PGE2 is not changed with prior incubation in EP2R or EP3R antagonist. **(E)** CD-1 mice respond with biphasic change in XII burst frequency to PGE2, while C57BL/6 WT and EP3 knock out mice only show an increase in their frequency across the concentration range tested **(F)** Change in XII burst period of CD-1 WT mice in response to 10 nM PGE2 is different from C57BL/6 WT (–14% below baseline, –39% below CD-1 control, *p* < 0.0001 vs. control) and Ptger3-/- knockouts (–11% below baseline, –36% below CD-1 control, *p* < 0.0001 vs. control), but not at 1 μM PGE2 (*p < 0.05, **p < 0.01, ****p < 0.0001).

The biphasic dose response in inspiratory rhythm frequency might be explained by differences in receptor binding affinity among Prostaglandin E2 receptors (Table 1). We attempted to distinguish the relative contributions of the EP receptor subtypes by utilizing selective antagonists while exposing rhythmic slices to PGE2 (10 nM or 1 μM, Figure 3 and Supplementary Table 3). Following a baseline recording, we preincubated each slice with EP1-4, FP, or DP2 receptor antagonists for 10 min before bath application of PGE2 (Figure 3A). For all the antagonists that we tested, the duration of the inspiratory burst period did not change in the presence of the antagonist by itself (Supplementary Table 4). Inhibiting EP2R attenuated the change in burst period normally induced by in both 10 nM PGE2 (−132% effect size compared to uninhibited controls) and 1 μM PGE2 (−100% effect size, Figure 3B). Inhibition of EP3R also attenuated the change in burst period at both 10 nM PGE2 (−72% effect size) and 1 μM (−64% effect size, Figure 3B). In contrast, inhibiting EP1 or EP4 receptors did not modify the biphasic response to PGE2. Neither XII burst area or variability was affected by incubation with PGE2 and antagonists (Figures 3C,D). Higher PGE2 concentrations (e.g., 1 μM) might additionally stimulate prostanoid receptors like DP2 and FP, which have dissociation constants in the sub-micromolar range (Table 1). In slices pre-incubated with a selective DP2 receptor antagonist (Setipiprant), the inspiratory burst period did not change after bath application of 10 nM PGE2 but it did decrease as expected in response to 1 μM. These results suggest that binding of EP2, EP3, and DP2 receptors is necessary to lengthen the burst period in 10 nM PGE2, whereas only EP2 and EP3 receptors are necessary to shorten the burst period in 1 μM PGE2.

Finally, we attempted to measure the response to PGE2 in mice that do not express EP3 receptors. We recorded inspiratory-related motor output in acute slices taken from transgenic Ptger3^–/–^ knock-out mice. These Ptger3^–/–^ mice were bred on a C57BL/6 background strain, which was different from the CD-1 wildtype mice used for our other experiments. The inspiratory burst period in both C57BL/6 wildtype controls and Ptger3^–/–^ knockouts did not increase in 10 nM PGE2 as it did with CD-1 mice (Figures 3E,F). In 1 μM PGE2 the inspiratory burst period decreased in both C57BL/6 wildtype (−25% below baseline) and Ptger3^–/–^ knockouts (−33% below baseline Figure 3F). This suggest that the stimulatory effect of PGE2 at 1 μM is conserved between CD-1 and C57BL/6 mice, and that EP3 receptors may not be developmentally necessary for 1 μM PGE2 to modulate inspiratory rhythm. However, the depressive effect of 10 nM PGE2 was not consistent between these two mouse strains, which could mean that there are strain-related differences in the sensitivity of the preBötC to low concentrations of PGE2.

### EP2R and EP3R Agonists Are Sufficient to Modulate Inspiratory Rhythm

**FIGURE 4:**
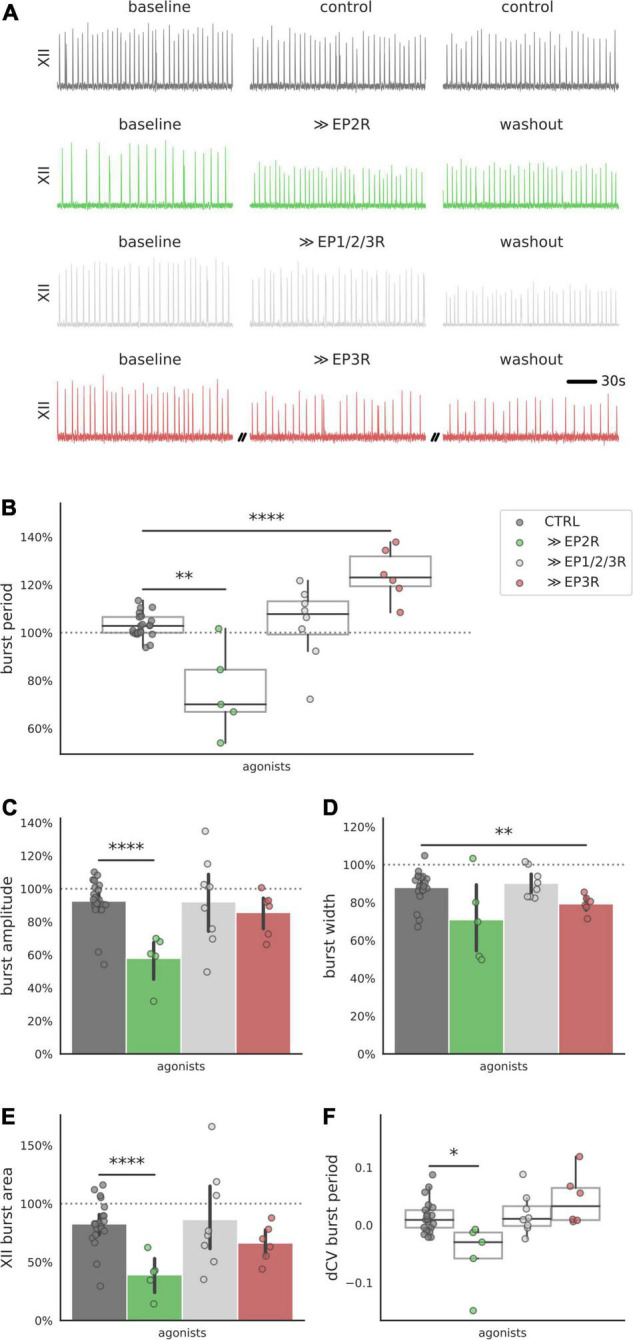
Integrated XII motor output in preBötC slices after exposure to prostanoid receptor agonists. **(A)** Examples of integrated hypoglossal motor output (XII) of slices exposed to EP2R agonist Butaprost (500 nM), EP3R agonist Sulprostone (1 μM) and non-selective prostanoid receptor agonist 11-deoxy PGE1 (10 nM) (150 s shown per condition). **(B)** Activating prostanoid receptors with selective agonists modulates burst period of XII motor output. Exposure to EP2R agonist Butaprost decreases burst period (–28% below control, *p* < 0.01), while EP3R agonist Sulprostone increases burst period (+21% above control, *p* < 0.0001). Non-selective prostanoid receptor agonist 11-deoxy PGE1 does not change the burst period. **(C)** EP2R agonist Butaprost decreases average burst amplitude (–35% below control, *p* < 0.001). **(D)** EP3R agonist Sulprostone decreases burst width (–9% below control, *p* < 0.01). **(E,F)** EP2R agonist Butaprost decreases XII burst area (–44% below control, *p* < 0.001) and the CV of the burst period (–0.07 below control, *p* < 0.05) (*p < 0.05, **p < 0.01, ****p < 0.0001).

Antagonizing a single receptor with specific pharmacology is useful in identifying receptor subtypes that are necessary for the biphasic PGE2 response. However, the responses we measured during receptor blockade could arise from mixed activity among the multiple prostanoid receptors that were left uninhibited. To better discriminate the actions of EP2 and EP3 receptors, we used specific agonists to stimulate EP2 and EP3 receptors independently (Figure 4A). Application of the EP2R agonist Butaprost shortened the burst period (−25% below baseline, Figure 4B). The EP3 receptor agonist, Sulprostone, lengthened the burst period (+24% above baseline, Figure 4B). A non-selective agonist that binds EP1R, EP2R, and EP3R (11-deoxy PGE1) did not change the burst period (Figure 4B). EP2R activation also decreased burst amplitude (−42% below baseline, Figure 3C), and EP3R activation decreased burst width (−21%, Figure 4D). XII burst area and the CV of the burst period also decreased after application of EP2R agonist Butaprost (area −61% below baseline, Figures 4E,F. These results suggest that biased activation of EP2 receptors is sufficient to shorten the inspiratory burst period, while biased activation of EP3 receptors is sufficient to lengthen the inspiratory burst period.

### Single-Cell Transcript Abundances of EP Receptor Subtypes

**FIGURE 5:**
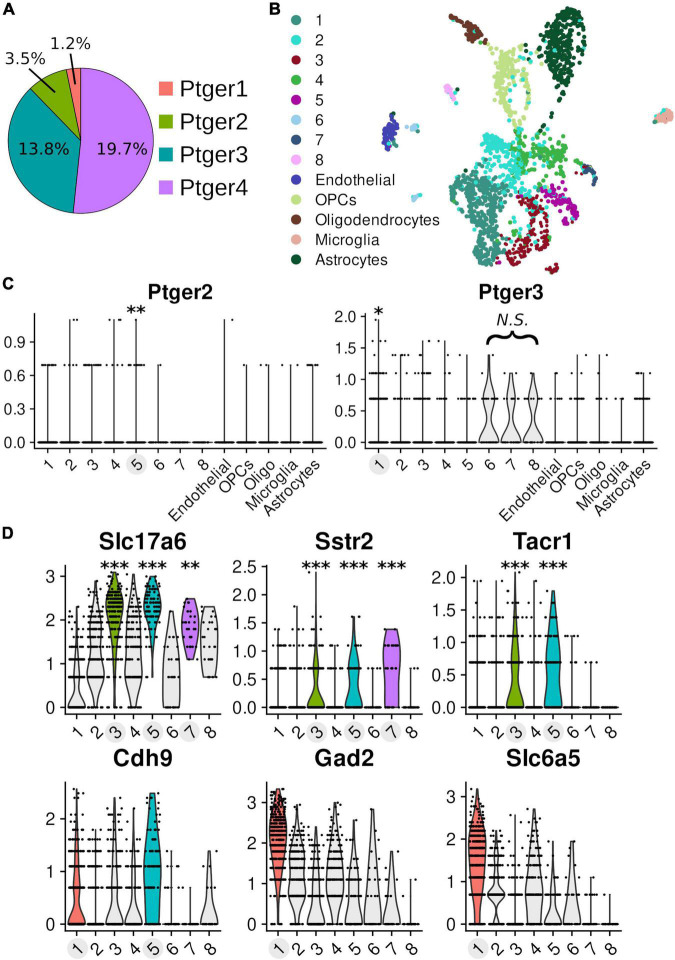
Single-cell transcriptomics of pre-Bötzinger complex cells Transcriptomic data of 2119 cells from microdissected tissue punchouts targeted at the pre-Bötzinger complex (*n* = 10, age post-natal day 4). On average, 11,247 unique molecules and 4,898 genes were detected per cell. Clusters with differential expression have labels highlighted in light gray and the associated violin distributions of significant expression are rendered in color. **(A)** Proportion of prostanoid receptor transcripts detected across all cells in the dataset: *Ptger4 n* = 418 cells, *Ptger3 n* = 292 cells, *Ptger2 n* = 73 cells, and *Ptger1 n* = 26 cells. **(B)** Dimensional reduction of the data in a 2D UMAP visualization, with the results of clustering into 13 groups overlaid by color. **(C)** Normalized expression values (*y*-axis) for EP2 receptors (*Ptger2*) and EP3 receptors (*Ptger3*) across different clusters. Differential expression for *Ptger2* in cluster 5 (log2 fold-change: 0.134, 12.4% of cells in cluster vs. 3.0% other cells, *p* = 0.00698). In cluster 1, there was differential expression for *Ptger3* (log2 fold-change: 0.154, 21.3% of cells in cluster vs. 11.9% other cells, *p* = 0.0185) and *Ptger4* (log2 fold-change: 0.267, 30.9% of cells in cluster vs. 17.0% other cells, *p* = 5.15e-6). **(D)** Markers for glutamatergic (*Slc17a6*), GABAergic (*Gad2*), and glycinergic (*Slc6a5*) neurons. Markers that overlap with the core of the preBötC: SST receptors 2 (*Sstr2*), neurokinin-1 (NK1R; *Tacr1*), cadherin-9 (*Cdh9*) (*p < 0.05, **p < 0.01, ***p < 0.001). See Supplementary Table 2 for values.

PreBötC slice recordings of hypoglossal motor output cannot readily distinguish which cell populations contained in the slice mediate the acute effects of PGE2 on inspiratory burst period. We therefore measured the relative abundances of the gene transcripts corresponding to prostanoid receptors (*Ptger1*-*4, Ptgdr2*, and *Ptgfr*) in single-cell RNA-Seq libraries of cells (*n* = 2119) that were isolated from punchouts of brainstem slices containing the preBötC (*n* = 10 pooled samples from littermates, age postnatal day 4, single 9600-microwell PCR plate). On average, we detected 11,247 unique molecules and 4,898 genes per cell. We detected transcripts for *Ptger4* in 418 cells, *Ptger3* in 292 cells, *Ptger2* in 73 cells, *Ptger1* in 26 cells, and *Ptgfr* in 48 cells (Figure 5A). We did not find any cells expressing transcripts for DP2 receptors (*Ptgdr2*). Clustering of the data partitioned cells into 13 groups (Figure 5B). Using cell-type-specific markers, we identified individual clusters that differentially expressed gene transcripts specific to astrocytes, microglia, oligodendrocytes, oligodendrocyte precursor cells, and endothelial cells (McKenzie et al., 2018; Supplementary Figure 1). Each of the remaining 8 clusters differentially expressed *Stmn2*, a neuron-specific marker, when compared to cells from non-neuronal clusters (Supplementary Table 1). We found two clusters that differentially expressed EP-receptor transcripts. Neuronal cluster 1 differentially expressed *Ptger3* and *Ptger4* (*Ptger3*: *p* = 0.018; *Ptger4*: *p* = 5.15e-6; Figure 5C). Cluster 1 was also the only cluster to differentially express GABAergic (*Gad2*, *p* = 5.3e-123; *Slc32a1*, *p* = 2.46e-128; Figure 5D and Supplementary Table 2) and glycinergic markers (Slc6a5, *p* = 1.99e-95; Figure 4D and Supplementary Table 2). Neuronal cluster 5 differentially expressed Ptger2 (*p* = 0.007; Figure 4C), and was also the only cluster with overlapping differential expression for Slc17a6 (VGLUT2), Tacr1 (NK1R), Sstr2 (SST receptor 2), and Cdh9 (Cadherin-9) (Figure 5D and Supplementary Table 2). These data suggest that, within the anatomical vicinity of the preBötC, cells expressing EP3 and EP4 receptors are generally more abundant than cells expressing EP1 and EP2 receptors. EP3 and EP4 receptors may be most enriched among inhibitory neurons, but our data cannot distinguish what proportion of EP3R^+^/EP4R^+^ inhibitory neurons are constituents of the preBötC rhythm-generating network. Finally, despite being detected in only 3.5% of all cells, transcripts for EP2 receptors were enriched among excitatory neurons that also differentially express markers known to overlap with the core of the preBötC (Gray et al., 2010; Gray, 2013; Yackle et al., 2017).

## Discussion

In rhythmically active slices containing the preBötC, inspiratory rhythm has a biphasic response to increasing concentrations of PGE2. Nanomolar concentrations of PGE2 (1–10 nM) increase both the irregularity and mean duration of the inspiratory burst period. Conversely, micromolar concentrations (1 μM) shorten the duration of the burst period. Selectively activating individual prostanoid receptors supports our hypothesis that opposing responses to PGE2 result from differences in receptor binding affinity. Stimulating EP3 receptors, which have the highest affinity for PGE2, also lengthens the duration of the burst period. In contrast, stimulating EP2 receptors, which have a lower affinity for PGE2, shortens the burst period. However, antagonizing either EP2 receptors or EP3 receptors abolishes the effect of PGE2 at any concentration. This suggests that the receptors have some degree of codependence—they work together to modulate respiration.

The present *in vitro* experiments demonstrate that changes in inspiratory burst period caused by PGE2 are concentration-dependent. This might mean that the response to endogenously released PGE2 *in vivo* also is biphasic. However, the concentrations we tested could also be non-physiological, over- or undershooting the typical bioavailability of PGE2 when it is released as an endogenous neuromodulator or during inflammatory events. PGE2 measured in cerebrospinal or amniotic fluid serves as a proxy for local concentrations in the brain and ranges from 1 to 50 nM following inflammatory events (Nilsberth et al., 2009; Engström et al., 2012; Westover et al., 2012). PGE2 sampled directly from brain tissue was consistently found in concentrations of approximately 50 pM across several brain regions after administering IL-1β (Komaki et al., 1992). Endogenous release of PGE2 in the preBötC might therefore lead to similar physiological concentrations. This would also agree with previous findings in newborn rats, mice, sheep, and human infants (Mitchell et al., 1978; Olsson et al., 2003; Hofstetter et al., 2007; Siljehav et al., 2015; Forsberg et al., 2016; Alvaro and Rigatto, 2018; Stojanovska et al., 2022).

Prostaglandin E2 does not change the burstlet fraction. Since we measured only XII motor output (i.e., not paired with integrated preBötC recordings), we inferred the burstlet fraction by analyzing the distribution of the burst periods before and after PGE2 exposure (Mellen et al., 2003; Sun et al., 2019). Depending on whether it modulates rhythm- or patter-generating neurons, PGE2 could either change the period of the burstlets or the proportion of burstlets that trigger a burst. If the burstlet fraction is changed, the distribution should become multimodal after PGE2, with each mode corresponding to a multiple of the burstlet period. In our experiments, the burst periods after exposure to PGE2 were unimodally distributed and periods did not cluster at multiples of the baseline period (Figure 2). Thus, PGE2 likely modulates rhythm-generating instead of pattern-generating processes in the preBötC. Moreover, burst pattern (e.g., burst width, amplitude, or area) did not change as a result of PGE2 at either low or high concentrations. We observed a gradual decline in burst height and burst width across all our slice recording experiments, including untreated control experiments. However, the mean duration of the burst period did not naturally run down in our experiments, which suggests that the decline in burst size might be attributed to a loss in signal-to-noise rather than a break-down of inspiratory rhythm generation. Although we took care preserving the hypoglossal rootlets in our recordings, it is possible that the health of the rootlets was routinely suboptimal or that our suction electrodes failed to maintain a constant resistance, which could weaken burst size (but not frequency) over time.

Here we focused mainly on EP1-4 receptors, but PGE2 can potentially activate DP2 and FP receptors. Inhibiting FP receptors had no influence on the response to PGE2, but DP2 receptor inhibition abolished the depressive effect of 10 nM PGE2. This was surprising because we did not expect DP2 receptors to be strongly activated by 10 nM PGE2. The K_*i*_ of DP2 receptors is 307 nM—more than 30-fold the tested concentration of PGE2. Setipiprant should be highly selective for DP2 receptors (Fretz et al., 2013). However, we do not know if the literature-reported specificity is maintained in the microenvironment of the acute slice. In addition, mRNA transcripts for DP2 receptors (*Ptgdr2*) were not detectable in any cells in our scRNA-Seq data. Most likely, DP2 receptors are not expressed, and Setipiprant has some off-target interactions that confounded the experiment. However, the absence of *Ptgdr2* transcripts might indicate that DP2 receptors are found in regions outside the anatomical vicinity of the preBötC (i.e., the boundaries of our tissue punchouts). Other neuronal populations contained in the rhythmic slice, like the raphe obscurus, can modulate activity in the preBötC (Ptak et al., 2009), and these neurons might yet express DP2 receptors, which would not be detected via our scRNA-Seq sampling strategy. Therefore, some minor fraction of DP2 receptors may yet need to be stimulated for inspiratory burst periods to become longer in 10 nM PGE2. Additional immunohistology or mRNA *in situ* hybridization assays could help resolve whether DP2 receptors are found in other preBötC-adjacent populations.

In the analysis of our scRNA-Seq data we found two clusters of cells that differentially expressed mRNA encoding for EP receptors–cluster 1 (*Ptger3* and *Ptger4*) and 5 (*Ptger2*).

Cluster 5 consists of glutamatergic neurons that express known markers for the preBötC: *Tacr1*, *Sstr2*, and *Cdh9* (Gray, 2013; Yackle et al., 2017). The overlap of these markers strongly supports that these are excitatory neurons *located* in the preBötC, but the data cannot distinguish whether cells in cluster 5 are part of the rhythm-generating population. If neurons in cluster 5 are excitatory and express EP2 receptors, then PGE2 likely stimulates rhythm-generation in the preBötC through these cells. EP2 receptors preferentially couple to Gs subunits whose downstream activity increases intracellular cAMP (Honda et al., 1993; Regan et al., 1994). Increasing intracellular cAMP in the preBötC increases inspiratory frequency through PKA-dependent phosphorylation of postsynaptic AMPA receptors (Shao et al., 2003). If EP2 receptors are differentially expressed in excitatory preBötC neurons, biased activation of EP2 receptors ought to increase tonic excitability in the preBötC, causing rhythmic activity to become faster. In support of this, we found that stimulating EP2 receptors with a selective agonist reduced the duration of the inspiratory burst period (Figure 4A).

Cluster 1 is a mixed group of GABAergic and glycinergic neurons. Unlike cluster 5, cluster 1 does not differentially express preBötC-specific markers compared to other neurons in the data. Therefore, we cannot determine what proportion of inhibitory neurons in cluster 1 are part of the preBötC versus some neighboring population (e.g., the Bötzinger complex). Nonetheless, we can speculate how EP3 receptor activation on inhibitory preBötC neurons might influence inspiratory burst frequency. EP3 receptors have different isoforms that couple to different G subunits (Namba et al., 1993; Negishi et al., 1993, 1995; Sugimoto et al., 1993). In the preBötC, EP3γ, which couples to Gs, is the most abundant isoform, followed by EP3α, which couples to Gi (Forsberg et al., 2016). Inhibitory activity that occurs during the inspiratory phase can increase preBötC burst frequency by reducing the duration of the post-burst refractory period (Baertsch et al., 2018). Inhibitory activity that occurs during the expiratory phase prolongs the onset of the next burst, decreasing the frequency of activity (Sherman et al., 2015). If EP3γ receptors are expressed on expiratory neurons, then PGE2 should increase intracellular cAMP, stimulate expiratory phase activity, and extend the duration of the inspiratory burst period. Alternatively, if EP3α receptors are expressed in inhibitory neurons that fire during the inspiratory phase, then PGE2 should reduce inspiratory inhibition, extend the duration of the refractory period, and slow preBötC burst frequency. In this case, we might also expect to see an increase in burst width and amplitude, since excitatory activity is less constrained.

There are several limitations with our experimental design and data. Rhythmic slice preparations do not perfectly isolate the preBötC. Neighboring populations, such as serotonergic neurons in the raphe obscurus, or reciprocal projections from the NTS and downstream premotor neurons might be sensitive to PGE2 (Ptak et al., 2009; Yang et al., 2020). If so, these neurons could indirectly modulate activity in the preBötC, and thereby confound our interpretation of the data, which partly relies on a paired analysis of transcriptomic data derived from cells that closely encompass the preBötC. Indeed, DP2 receptor mRNA does not appear in our scRNA-Seq data but inhibiting DP2 receptors attenuates the depressive effect of 10 nM PGE2. This might be explained by technical artifacts (e.g., off target pharmacology or failure to capture mRNA) or by DP2-sensitive neurons in other parts of the slice.

Conversely, slice preparations are deafferented from rostral regions of the ventral respiratory column, including the Bötzinger complex, parafacial respiratory group, and postinspiratory complex. These regions provide feedback to the preBötC during postinspiratory and expiratory phases of respiratory rhythm generation. Loss of these regions could explain why PGE2 administered in other, more intact, contexts consistently depresses breathing, while not in acute slices. We argue that lower concentrations of PGE2 more faithfully approximate physiological release events because 1–10 nM PGE2 elongates the inspiratory burst period in our experiments. That is, a stimulus of lesser magnitude recapitulates both *in situ* measurements of PGE2 concentration and the behavior observed *in vivo*. This is consistent with the physiological data regarding concentrations in the pico to nanomolar range and the effects of exogenous PGE2 administration *in vivo* to mice, rats, rabbits, sheep and humans. However, neuromodulatory actions in the brain can be both activity and state-dependent (Marder et al., 2014; Burgraff et al., 2021; de Sousa Abreu et al., 2022). The behaviors we observe in deafferented slices *in vitro* could resemble *in vivo* responses but nonetheless arise through different mechanisms due to different modulatory status and loss of inputs.

In contrast to the CD-1 mice used in our other experiments, the burst period in C57BL/6 wildtype mice did not get longer in 10 nM PGE2. Instead, the response to PGE2 was monophasic—the burst period became shorter when the concentration of PGE2 increased to 1 μM. This could mean that C57BL/6 mice are less sensitive to the inhibitory effects of 10 nM PGE2 that we originally observed in CD-1 mice. Indeed, there are documented differences in breathing behavior observed in CD-1 mice versus C57BL/6 mice. Notably, breathing is less stable in early neonatal CD-1 mice. At P0, CD-1 mice have frequent apneas that gradually stabilize between P1 and P3, while C57BL/6 mice do not exhibit apneas under normoxic conditions between P1-P3 (Robinson et al., 2000; Bissonnette and Knopp, 2001). However, we also know that inspiratory-related output slows down after PGE2 exposure in brainstem spinal cord *en bloc* preparations taken from C57BL/6 wildtype mice, and the effect is abolished in *Ptger*^–/–^ mice (Hofstetter et al., 2007). Therefore, slice preparations taken from C57BL/6 mice may be insensitive to 10 nM PGE2 because of strain related differences, or because of lost afferents in the acute slice, or perhaps due to some other uncontrolled state in the experiments. For instance, owing to the limited number of recordings per condition, we could not stratify our data by postnatal age. But in rats and sheep, sensitivity to PGE2-induced apnea is strongest immediately at birth and levels off with postnatal age—as early as P2-P4 in rats and P15 in sheep (Tai and Adamson, 2000; Ballanyi, 2004). Thus, it is plausible that the different responses to PGE2 between strains reflect variation in early postnatal development. Additional experiments involving more granular age-matched comparison between CD-1 and C57BL/6 mice would be necessary to test this point.

Differential expression testing in our transcriptomics data has allowed us to speculate how EP2 and EP3 receptors might modulate the frequency of inspiratory activity in the preBötC. But *Ptger3* and *Ptger2* expression was sparse in our data overall (Figure 5). Even in clusters 1 and 5, where the receptors were differentially expressed, *Ptger3* and *Ptger2* are found in only 21 and 12% of the clustered cells, respectively. Although these proportions are not very high, small numbers of neurons can certainly modulate the frequency of inspiratory rhythm. For instance, as few as 70–140 neurons putatively mediate opioid sensitivity in the preBötC (Bachmutsky et al., 2020). We nonetheless advise caution when drawing conclusions from our scRNA-seq data for two reasons. First, the dataset consists of a single technical replicate and lacks *in situ* validation. Although we sampled cells from several (*n* = 10) neonatal mouse pups, all samples were prepared on the same day and cell libraries were produced on a single 9600-microwell chip. This means we were unable to statistically model and compensate for the effect of methodological bias or batch-to-batch technical noise in our data. Batch effects in scRNA-seq data are a well-known and significant source of bias that could influence the results of cell clustering or differential expression testing, particularly for sparsely expressed genes like *Ptger3* and *Ptger2*. Second, the resolution of our data is limited both in terms of absolute cell numbers and the number of detected transcripts. Given that prostanoid receptor expression is low-level and sparse, the effect sizes in differential expression testing are sensitive to cell filtering thresholds and clustering resolution. As such, we refrained from using strict thresholds, attempting to retain as many cells as possible, and otherwise used common parameter defaults during dimensionality reduction and clustering. In the future, additional experiments involving more technical replicates, multiplexed *in situ* hybridization, higher sequencing depth, and more sensitive library preparation methods could help solidify and extend the work presented here.

Taken together our data demonstrate that inspiratory-related motor rhythm can have a biphasic response to increasing concentrations of PGE2. We predicted that lower concentrations of PGE2 favor activation of high affinity receptors, like EP3R, that slows inspiratory activity. Higher concentrations of PGE2 favor activation of low affinity receptors, like EP2R, that accelerates inspiratory activity. We found that the biphasic response is mediated through EP2 and EP3 receptors. In line with our hypothesis, experiments with pharmacological experiments demonstrated that preferential activation of EP3 receptors slows inspiratory activity, while EP2 receptors accelerate inspiratory activity. We have further identified candidate target neuronal populations that differentially express EP2R (excitatory preBötC) and EP3R (inhibitory). PGE2 can interfere with ventilatory chemoreflexes in newborns, worsening the ability to cope with environmental stress. Thoroughly understanding how PGE2 modulates breathing behavior will aid the pursuit of novel therapeutics that could rescue at-risk newborns from respiratory failure.

## Data Availability Statement

The original contributions presented in the study are publicly available. This data can be found here: NCBI, under accession number GSE192680.

## Ethics Statement

The studies were performed in accordance with European Community Guidelines and approved by the Stockholm Animal Research Ethics Committee (approval no. 15819-2017).

## Author Contributions

JR and WP designed and performed the *in vitro* electrophysiology experiments and wrote and revised the manuscript. JR analyzed the *in vitro* electrophysiology data. WP and NR prepared samples for scRNA-Seq experiments. WP analyzed and interpreted the scRNA-Seq data. EH conceptualized the project, provided funding and laboratory facilities, and revised the manuscript. All authors read and approved the final version of the manuscript.

## Conflict of Interest

The authors declare that the research was conducted in the absence of any commercial or financial relationships that could be construed as a potential conflict of interest.

## Publisher’s Note

All claims expressed in this article are solely those of the authors and do not necessarily represent those of their affiliated organizations, or those of the publisher, the editors and the reviewers. Any product that may be evaluated in this article, or claim that may be made by its manufacturer, is not guaranteed or endorsed by the publisher.
