# Supplementary material for: Prostaglandin E2 Exerts Biphasic Dose Response on the PreBötzinger Complex Respiratory-Related Rhythm
Source: Front Neural Circuits. 2022 May 20;16:826497. doi: 10.3389/fncir.2022.826497 (PMC9163299; doi:10.3389/fncir.2022.826497)

Supplementary Material

Supplementary Table 1: Stmn2 *expression in putative neuronal clusters*

Cluster	Log_2 F.C.	Adj. p-value
1	0.582	4.80E-82
2	0.445	1.97E-34
3	0.677	3.89E-59
4	0.346	1.32E-15
5	0.507	2.27E-23
6	0.537	9.24E-06
7	0.477	4.05E-05
8	0.976	9.11E-14

Note: Stmn2 was detected in 100% of cells.

Supplementary Table 2: Differential expression of transmitters and pre-BötC markers

	Log_2 F.C.	% Cluster	% All other cells	Adj. p-value
Slc17a6				
3	1.321	96.7	76.1	2.00E-69
5	1.410	100	77.4	6.50E-40
7	0.678	100	78.2	7.05E-03
Sstr2				
3	0.257	27.7	13.7	3.25E-04
5	0.410	42.9	13.9	4.06E-11
7	0.788	63.3	14.6	1.62E-10
Tacr1				
3	0.393	39.7	17.4	4.02E-12
5	0.586	54.3	18.2	7.33E-16
Cdh9				
1	0.282	42.1	29.2	9.27E-03
5	1.069	72.4	29.6	2.95E-20
Gad2				
1	1.542	98.1	75.7	5.30E-123
Slc32a1				
1	1.327	93.1	44.4	2.46E-128
Slc6a5				
1	1.382	90.4	53.4	1.99E-95

Supplementary Table 3: Statistical differences in respiration-related XII burst period of slices exposed to PGE2 compared to baseline for different prostanoid receptor antagonists

Antagonist	PGE2	Burst period (s)	estimate	SE	z.ratio	p.value
-	baseline	5.23 ± 1.52				
EP1R (GW848)	10nM	6.18 ± 2.65	-0.95	0.32	-2.960	**0.01**
	1µM	4.08 ± 1.30	1.15	0.21	5.490	< **0.001**
-	baseline	5.78 ± 1.33				
EP2R (PF044)	10nM	5.24 ± 1.32	0.54	0.31	1.767	0.18
	1µM	5.68 ± 1.56	0.10	0.39	0.269	0.96
-	baseline	5.02 ± 2.08				
EP3R (DG041)	10nM	6.13 ± 4.15	-1.11	0.62	-1.806	0.17
	1µM	4.69 ± 1.94	0.33	0.46	0.723	0.75
-	baseline	6.87 ± 3.13				
EP4R (MK289)	10nM	7.34 ± 4.22	-0.47	0.86	-0.554	0.84
	1µM	4.71 ± 1.07	2.16	0.69	3.133	**0.005**

Supplementary Table 4: Statistical differences of respiration-related XII burst period between baseline and after incubation in respective prostanoid receptor antagonists

Receptor	condition	Normalized burst period	estimate	SE	z.ratio	p.value
CTRL	-	1.03 ± 0.05	-	-	-	-
EP1R	GW-848867X	1.16 ± 0.25	-0.12	0.075	-1.66	0.96
EP2R	PF-04418948	0.94 ± 0.14	0.09	0.061	1.45	1.00
EP3R	DG-041	1.02 ± 0.08	0.01	0.022	0.54	1.00
EP4R	MK-2894	1.16 ± 0.14	-0.13	0.048	-2.75	0.12
DP2	Setipiprant	0.94 ± 0.19	-0.12	0.085	1.11	1.00
FP	AL-1180	1.09 ± 0.26	-0.06	0.069	-0.88	1.00

Supplementary Figure 1: Expression of major cell type markers

Markers for major cell types used to annotate clusters: astrocytes (*Aqp4*), microglia (*Cx3cr1*), oligodendrocytes (*Mbp*), oligodendrocyte precursor cells (*Pdgfra*), endothelial cells (*Cd34*), and neuronal-like cells *(Stmn2*). See also Supplementary Table 1.

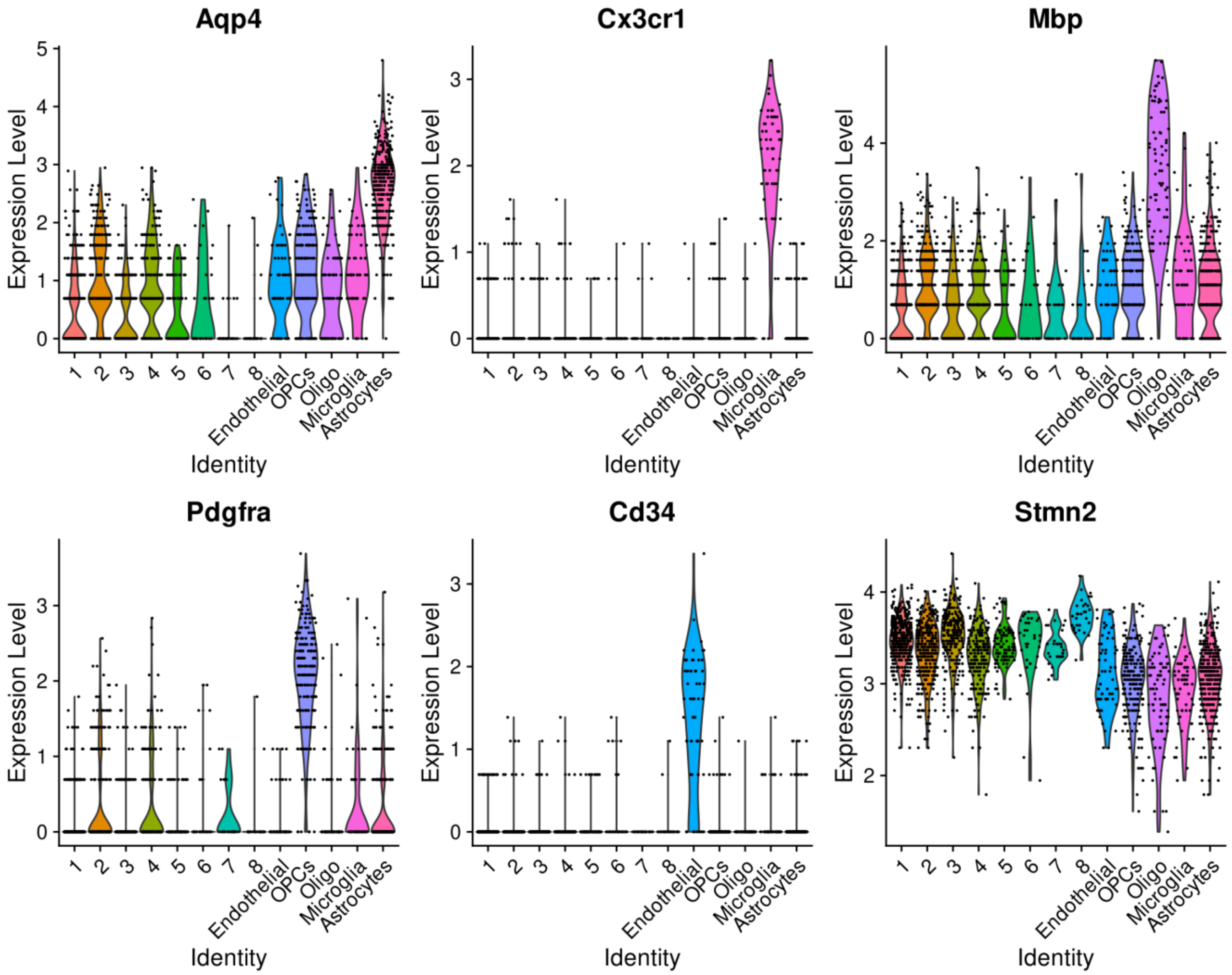

Supplement: Supplementary file 1 [file Data_Sheet_1.pdf]
